# Epidemiology, evolution, and biological characteristics of avian influenza A (H11) viruses from wild birds

**DOI:** 10.1080/21505594.2025.2591462

**Published:** 2025-11-19

**Authors:** Zhiguo Zhao, Jingman Tian, Xiaoli Bai, Minghui Li, Xingdong Song, Jiaying Li, Jianzhong Shi, Huihui Kong, Xianying Zeng, Guobin Tian, Jinxiong Liu, Chengjun Li, Hualan Chen, Yanbing Li

**Affiliations:** State Key Laboratory for Animal Disease Control and Prevention, Harbin Veterinary Research Institute, Chinese Academy of Agricultural Sciences, Harbin, People’s Republic of China

**Keywords:** Avian influenza virus, H11 subtype, wild birds, phylogeny, receptor binding specificity, pathogenicity

## Abstract

H11 subtype avian influenza viruses (AIVs) have been identified in both wild and domestic birds. H11N9 viruses from wild birds provided the NA gene to human H7N9 virus in 2013 in China, which caused five waves of human infections. During active surveillance in wild birds in China, 17 H11 viruses were isolated between December 2022 and January 2024, including six H11N1, one H11N2, one H11N3, and nine H11N9. The epidemiology of H11 subtype viruses in public databases revealed that they distributed across seven continents, and more than 54.9% of H11 viruses originated from wild Anseriformes. Phylogenetic analysis of the HA genes indicated that H11 viruses were classified into Eurasian and North American lineages, and our isolates belonged to the Eurasian lineage. Bayesian phylogeographic analysis suggested that Bangladesh served as a crucial geographical transmission center for H11 viruses in Eurasian lineage. Reassortment indicated that the H11 isolates in the study underwent complex genomic recombination with various subtype AIVs circulating in wild and domestic birds, including the clade 2.3.4.4b H5N1 highly pathogenic viruses, and formed seven genotypes. Notably, 17 H11 isolates acquired several mutations associated with enhanced human-type receptor binding in HA (S137A) and increased mammalian virulence in PB1 (D3V, D622G), PB1-F2 (N66S), M1 (N30D, I43M, T215A), and NS1 (P42S, I106M). Seven representative viruses exhibited dual receptor binding specificity and could infect mice directly without prior adaptation. These findings highlight the potential public health risks posed by H11 viruses from wild birds and emphasize the necessity of enhancing routine surveillance.

## Introduction

Avian influenza viruses (AIVs), members of the genus influenza A virus in the family Orthomyxoviridae, are classified into multiple subtypes based on combinations of their surface glycoproteins: hemagglutinin (HA; H1–H16) and neuraminidase (NA; N1–N9) [1,2]. According to the pathogenicity in chickens and molecular characteristics of the HA cleavage site, AIVs are further categorized into highly pathogenic avian influenza (HPAI) viruses and low pathogenic avian influenza (LPAI) viruses [3,4]. In contrast to the well-recognized threat posed by HPAI viruses to poultry and public health, LPAI viruses can also cross species barriers and infect humans. Over the past several years, human infections with LPAI subtypes, such as H3N8, H6N1, H7N4, H7N9, H9N2, H10N3, H10N5 and H10N8, have been reported, indicating their zoonotic potential [5,6]. In our previous studies, we investigated the biological characteristics of H4N6 and H5N1 AIVs isolated from wild birds in China [7,8]. Building upon this body of work, the present study focuses on H11 subtype AIVs, which remain relatively understudied despite their widespread distribution and potential risk to both avian and mammalian hosts.

H11 subtype AIVs are classified as LPAI viruses and are geographically widespread [9]. In 1956, the first H11N6 avian influenza virus, A/duck/England/1956, was isolated from ducks in the United Kingdom [1]. Over the past 60 years, H11 AIVs have persisted in wild birds, with nine subtypes (H11N1–H11N9) identified. Wild birds, particularly waterfowl and shorebirds, are recognized as the primary natural reservoirs of AIVs [8,10]. Their seasonal migration between wintering and breeding grounds involves multiple stopovers along migratory flyways, providing opportunities for virus transmission to local waterfowl and facilitating intercontinental dissemination of AIVs [10]. Although H11 viruses are primarily transmitted among wild and domestic birds, the isolation of H11N6 from pigs and the detection of H11-specific antibodies in southern sea otters demonstrate the potential for the viruses to infect mammals [11,12]. Furthermore, Serological evidence of H11 virus exposure has also been reported among individuals frequently exposed to wild and domestic birds, including hunters, wildlife professionals, and Lebanese poultry workers [13,14]. Notably, the H11N9 virus has been identified as the donor of the NA gene of the H7N9, which caused 1,568 human infections with the fatality of 616, and posed a serious threat to public health [15].

In this study, 17 H11 viruses were isolated from the Yancheng wetland reserve in Jiangsu Province, China. We employed these viruses to investigate their genetic evolution, molecular characteristics, receptor-binding properties and virulence in mice. Our findings suggest that H11 AIVs may pose a potential public health risk, emphasizing the critical need to enhance the surveillance of H11 viruses in wild birds.

## Materials and methods

### Ethics statements and facility

All experiments involving animals were carried out in strict accordance with the recommendations in the Guide for the Care and Use of Laboratory Animals of the Ministry of Science and Technology of the People’s Republic of China. All studies involving live viruses were performed in a biosafety level 2 laboratory approved for such use by the Harbin Veterinary Research Institute (HVRI) of the Chinese Academy of Agricultural Sciences (CAAS). The animal experimentation protocol was approved by the Committee on the Ethics of Animal Experiments of HVRI (CAAS) under the reference GRLAIV(P2)-2024–025.

### Sample collection, virus isolation and identification

A total of 4,850 fecal samples were collected from wild bird habitats during routine environmental surveillance conducted by the HVRI of CAAS, between December 2022 and January 2024. Samples were non-invasively obtained from fresh droppings found in the environment, without capturing or disturbing the birds. Each sample was placed in a sterile Eppendorf (EP) tube containing phosphate-buffered saline (PBS) supplemented with 6,000 U/mL penicillin, 6,000 U/mL streptomycin, and 10% glycerol. The samples were inoculated into 10-day-old specific pathogen-free (SPF) chicken embryos and incubated at 37°C for 72 hours. Hemagglutination (HA)-positive allantoic fluids were collected, and the HA subtypes were identified using hemagglutination inhibition (HI) assays with a panel of H1–H16 reference sera. The NA subtypes were confirmed through direct sequence analysis.

### Genome sequencing

Viral RNA was extracted from HA-positive allantoic fluid using the QIAamp Viral RNA Mini Kit (QIAGEN, Germany) and reverse transcribed with Unit12 primers. Full-genome sequencing was performed using the 3500xL Genetic Analyzer (Applied Biosystems, USA). The nucleotide sequences were edited using the Seqman module of the DNAStar package. All generated nucleotide sequences were submitted to the GISAID EpiFlu™ database (https://www.gisaid.org).

### Epidemiology and genetic analysis

The HA sequences of global H11 subtype viruses and sample information were obtained on 14 March 2024, from NCBI GenBank and the GISAID EpiFlu™ database. After removing duplicate isolates, mixed-subtype isolates, and sequences with less than 85% gene coverage, a total of 1,003 H11 viruses were selected for further epidemiological and phylogenetic analysis. Statistical analysis was conducted in RStudio using the package ggplot2 v.3.4.1 [16].

Multiple sequence alignment was performed using MAFFT v.7.490 [17]. Maximum-likelihood (ML) trees were constructed using IQ-TREE v.1.6.12 [18] with the best-fit model and 1,000 ultrafast bootstraps. ML trees were visualized and annotated with the ggtree package v.3.7.1.003 [19]. Each gene segment in the ML tree was grouped based on a sequence homology criterion of greater than 95%.

### Bayesian phylodynamic analysis

The ML tree of HA sequences from Eurasian lineage H11 viruses was used to identify molecular clock outliers, using TempEst v.1.5.3 [20]. Divergence times and evolutionary rates were estimated under an uncorrelated relaxed clock model within a Bayesian framework using Markov chain Monte Carlo (MCMC) sampling in BEAST v.1.10.4 [21]. The MCMC chain was run for 200 million generations, sampling every 20,000 steps. Convergence was assessed in Tracer v1.7.2, and effective sample size (ESS) values above 200 were accepted. Finally, the maximum clade credibility (MCC) tree was generated using TreeAnnotator v1.10.4 with a 10% burn-in and visualized using the ggtree package v.3.7.1.003 [19].

To reconstruct spatial diffusion from Eurasian lineage H11 viruses, we conducted asymmetric discrete trait phylogeographic analyses across eight geographic regions (Africa, Bangladesh, China, Europe, Japan, Russia, Korea, Southeast Asia) and seven hosts (wild Anseriformes, wild Charadriiformes, Struthioniformes, Ciconiiformes, domestic Anseriformes, domestic Galliformes, Swine), using BEAST v.1.10.4 [21]. SpreadD3 v.0.9.6 was used to estimate Bayes factors (BF) for determining statistical significance, with migration pathways supported by a combination of BF ≥ 3 and posterior probability (PP) ≥ 0.5 [22]. Additionally, to account for all transitions between states and the time spent in states between transitions, we applied the continuous-time Markov chains model to complete the Markov jump history over time.

### Receptor binding assay

The solid-phase direct binding assay to test the receptor binding properties of the viruses has been described previously [23]. Seven representative viruses, corresponding to genotypes one to seven, were selected for the receptor binding assay. 96-well plates were coated with biotinylated α-2,3-sialylglycopolymer (avian-type receptor) and α-2,6-sialylglycopolymer (human-type receptor). Inactivated virus allantoic fluid, diluted to 64 HA units per well, were then added and incubated at room temperature. A specific chicken antisera (PFG/JS/4–2-314/2022 (H11N1)) was used as the primary antibody, and a horseradish peroxidase (HRP)-conjugated goat-anti-chicken antibody was used as the secondary antibody. The absorbance was measured at 450 nm, and each experiment was performed in triplicate.

### Animal experiments

Six-week-old female SPF BALB/c mice (Vital River Laboratories, Beijing, China) were randomly divided into seven inoculation groups (eight mice each) and one control group (five mice). The sample size was determined based on previous studies assessing the pathogenicity of AIVs in mice, with the aim of ensuring sufficient statistical power while minimizing animal use [7]. Cage position was randomized and procedures were conducted at the same time daily. The mice of inoculation groups were anesthetized via isoflurane inhalation and then inoculated intranasally with 10^6^ EID_50_ of the virus in a volume of 50 µL, respectively, with control group inoculated with 50 µL PBS. Three mice per inoculation group were euthanized on day 3 post-inoculation (p.i.), and brain, kidney, spleen, nasal turbinate and lung tissues were collected for viral titration in eggs using Reed-Muench method [24]. Viral titration was performed in a blinded manner, whereas group allocation was known to the investigators during animal inoculation and clinical monitoring. Mice were humanely euthanized if they exhibited > 25% body weight loss. The remaining five mice were monitored daily for 14 days for weight loss and mortality. On day 15 p.i., all surviving mice were anesthetized with isoflurane and humanely euthanized via CO_2_ asphyxiation, in accordance with the American Veterinary Medical Association (AVMA) guidelines for the euthanasia of animals. No inclusion or exclusion criteria were pre-specified for animals or data points in this study. All animals and collected data were included in the final analysis. Descriptive statistics (mean ± standard deviation) were used to summarize viral titers and body weight loss. Statistical analyses were performed using GraphPad Prism version 9.5.0 [25].

## Results

### 17 H11 viruses isolated from wild birds in Jiangsu Province, China, during 2022–2024

During routine surveillance of wild birds in Jiangsu Province, China, from December 2022 to January 2024, a total of 17 H11 viruses were identified and isolated from 4,850 samples (Table 1). These viruses were classified into four subtype combinations, including H11N1 (*n* = 6), H11N2 (*n* = 1), H11N3 (*n* = 1), and H11N9 (*n* = 9).

**Table 1.** H11 viruses isolated from wild birds in Jiangsu Province, China, during 2022–2024.

Date	Sample		H11 isolate			H11Nx		
	No.	Type	No.	Positive rate (%)	Subtype (No.)	Name	Abbreviation	Order
December 2022	1198	Feces	4	0.33	H11N1 (4)	A/bean goose/Jiangsu/1–1-451/2022(H11N1)	BG/JS/1–1-451/2022(H11N1)	Wild Anseriformes
						A/bean goose/Jiangsu/2–1-268/2022(H11N1)	BG/JS/2–1-268/2022(H11N1)	Wild Anseriformes
						A/bean goose/Jiangsu/3–1-207/2022(H11N1)	BG/JS/3–1-207/2022(H11N1)	Wild Anseriformes
						A/pink-footed goose/Jiangsu/4–2-314/2022(H11N1)	PFG/JS/4–2-314/2022(H11N1)	Wild Anseriformes
January 2023	1000	Feces	1	0.10	H11N2 (1)	A/mallard/Jiangsu/5–1-984/2023(H11N2)	ML/JS/5–1-984/2023(H11N2)	Wild Anseriformes
November 2023	973	Feces	3	0.31	H11N3 (1)	A/mallard/Jiangsu/6–2-54/2023(H11N3)	ML/JS/6–2-54/2023(H11N3)	Wild Anseriformes
					H11N9 (2)	A/graylag goose/Jiangsu/7–2-262/2023(H11N9)	GG/JS/7–2-262/2023(H11N9)	Wild Anseriformes
						A/graylag goose/Jiangsu/8–2-739/2023(H11N9)	GG/JS/8–2-739/2023(H11N9)	Wild Anseriformes
December 2023	1035	Feces	2	0.19	H11N1 (2)	A/graylag goose/Jiangsu/9–3-469/2023(H11N1)	GG/JS/9–3-469/2023(H11N1)	Wild Anseriformes
						A/graylag goose/Jiangsu/10–3-985/2023(H11N1)	GG/JS/10–3-985/2023(H11N1)	Wild Anseriformes
January 2024	644	Feces	7	1.09	H11N9 (7)	A/graylag goose/Jiangsu/11–1-57/2024(H11N9)	GG/JS/11–1-57/2024(H11N9)	Wild Anseriformes
						A/graylag goose/Jiangsu/12–1-198/2024(H11N9)	GG/JS/12–1-198/2024(H11N9)	Wild Anseriformes
						A/graylag goose/Jiangsu/13–1-337/2024(H11N9)	GG/JS/13–1-337/2024(H11N9)	Wild Anseriformes
						A/graylag goose/Jiangsu/14–1-591/2024(H11N9)	GG/JS/14–1-591/2024(H11N9)	Wild Anseriformes
						A/graylag goose/Jiangsu/15–1-594/2024(H11N9)	GG/JS/15–1-594/2024(H11N9)	Wild Anseriformes
						A/graylag goose/Jiangsu/16–1-603/2024(H11N9)	GG/JS/16–1-603/2024(H11N9)	Wild Anseriformes
						A/graylag goose/Jiangsu/17–1-611/2024(H11N9)	GG/JS/17–1-611/2024(H11N9)	Wild Anseriformes

### The prevalence of H11 subtype AIVs globally

**Figure 1.**
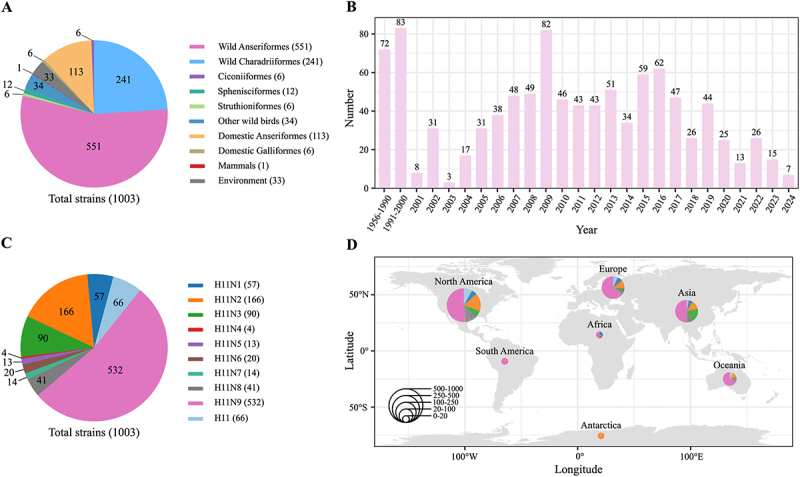
Prevalence of H11 subtype AIVs globally. (A) the proportion of the host of H11 virus. Hosts were categorized according to virus isolation information. (B) Temporal distribution of H11 virus detections from 1956 to 2024. (C) HA and NA combinations of H11 subtype AIVs. (D) Spatial distribution of H11 viruses across continents. Each pie chart represents the relative proportion of subtypes in each region, with chart size proportional to the number of isolates.

To better understand the epidemiology of H11 AIVs, we created a dataset of 1,003 HA sequences, including 17 strains isolated in this study and 986 sourced from NCBI GenBank and the GISAID database (Table S1). As shown in Figure 1(A), more than 84.7% of H11 viruses (*n* = 850) were isolated from wild birds, with the majority originating from wild Anseriformes (*n* = 551; 54.9%), followed by wild Charadriiformes (*n* = 241; 24.0%). Additionally, 119 isolates were obtained from domestic birds, primarily domestic Anseriformes (*n* = 113; 11.3%), and a smaller proportion from domestic Galliformes (*n* = 6; 0.6%). Of note, one isolate (A/swine/KU/2/2001) was derived from a swine host. Since the first detection of H11 viruses in 1956, these viruses have persisted in circulation, with a marked increase in detections observed between 2005 and 2019, peaking in 2009 (Figure 1(B)). In terms of subtypes, H11N9 was the most prevalent, accounting for 532 isolates, followed by H11N2 (*n* = 166; 16.6%) and H11N3 (*n* = 90; 9.0%) (Figure 1(C)). Geographically, H11 viruses were distributed worldwide, with the highest detection rates in North America (*n* = 625; 62.3%), followed by Asia (*n* = 192; 19.1%), and Europe (*n* = 109; 10.9%) (Figure 1(D)). These results highlight the broad host range and geographical distribution of H11 AIVs.

### Phylogenetics of Ha sequences of H11 viruses

**Figure 2.**
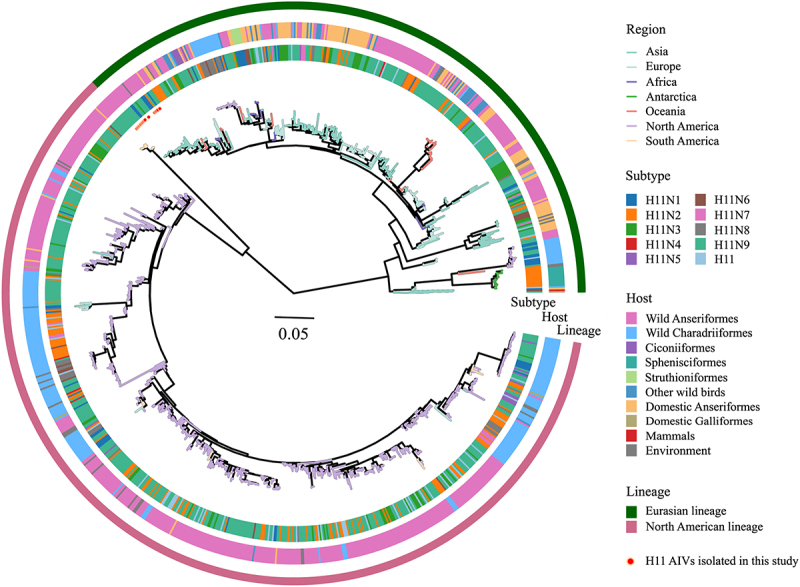
Maximum likelihood phylogenetic tree of ha genes of 1,003 H11 AIVs from 1956 to 2024. Virus categories (regions, hosts, and subtypes) are indicated by different colors. The 17 H11 viruses isolated in this study are marked with red circles. The tree was further visualized and edited using the ggtree package v.3.7.1.003.

To investigate the genetic evolution of H11 subtype AIVs globally, we conducted a phylogenetic analysis using the complete dataset of 1,003 H11 AIVs. Our findings indicate that global H11 viruses are classified into two distinct lineages, the Eurasian lineage and the North American lineage (Figure 2, Table S1). The 17 isolates in this study belonged to the Eurasian lineage. Noteworthy, the swine-origin isolate, A/swine/KU/2/2001, was also assigned to the Eurasian lineage.

**Figure 3.**
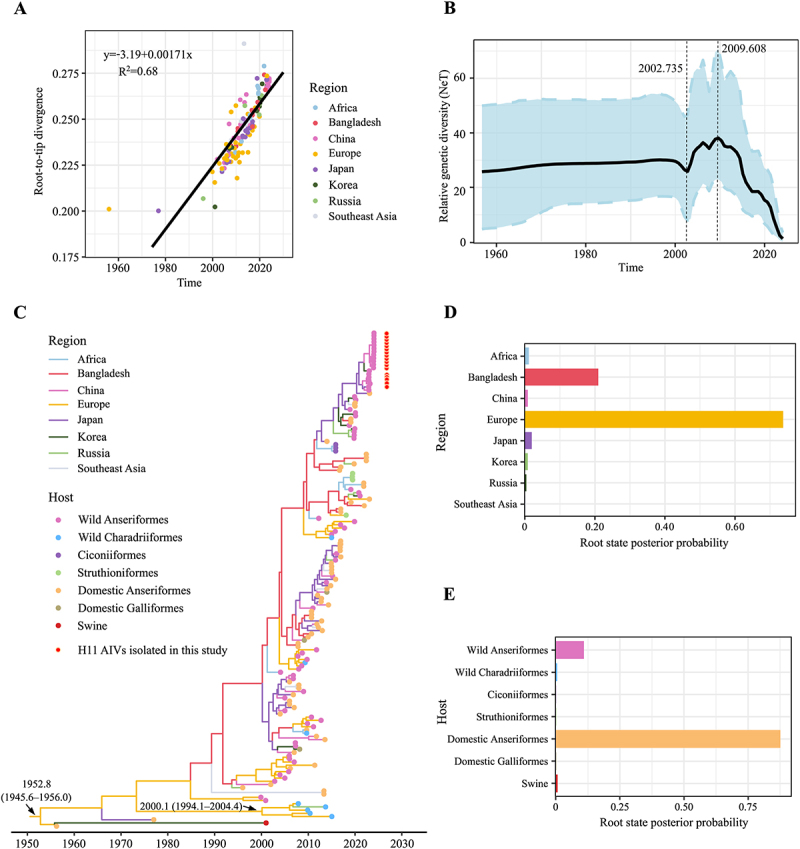
Bayesian phylodynamic analysis of ha genes of H11 viruses in the Eurasian lineage. (A) Bayesian skyline plot (bsp) showing relative genetic diversity over time. The black line represents the mean genetic diversity, and the light blue shaded area indicates the 95% confidence interval. (B) Root-to-tip regression analysis for temporal signal estimation. (C) Time-scaled mcc tree of H11 virus ha sequences in the Eurasian lineage, with branches colored according to different geographic regions. Colored dots at branch tips indicate various host species, and 17 isolates identified in this study marked by red circles. (D) Posterior probabilities of root state by region. (E) Posterior probabilities of root state by host. Plots (A), (B), (C), and (E) were generated using ggplot2 package v.3.4.1.

To assess the temporal signal of the HA gene of H11 viruses in Eurasian lineage, we constructed a ML tree and performed a root-to-tip regression analysis. The correlation coefficient and R^2^ values were 0.82 and 0.68, respectively, confirming a strong temporal structure (Figure 3(A)). Furthermore, the evolutionary rate of the HA gene was calculated to be 0.00171 substitutions/site/year. Using Bayesian skyline coalescent reconstruction, we assessed the relative genetic diversity of H11 AIVs to infer their effective population size. Before 2000, the population size of H11 AIVs from the Eurasian lineage remained relatively stable, followed by an expansion between 2002 and 2010 (Figure 3(B)). Interestingly, after a period of fluctuations, a significant decrease in population size was observed after 2010, suggesting a reduction in the relative genetic diversity of these viruses. The time-scaled MCC tree, based on the HA gene of Eurasian lineage H11 viruses, indicated that the time to the most recent common ancestor (TMRCA) dates back to 1952.8 (95% HPD: 1945.6–1956.0) (Figure 3(C), Figure S1). Additionally, wild Charadriiformes in the MCC tree evolved independently from a branch with a TMRCA of 2000.1 (95% HPD: 1994.1–2004.4). Bayesian analysis places the root of the trees in Europe with a posterior probability of 0.74 (Figure 3(D), Table S2), and domestic Anseriformes with a posterior probability of 0.88 (Figure 3(E), Table S3).

### Phylogeography and transmission of H11 viruses in the Eurasian lineage

**Figure 4.**
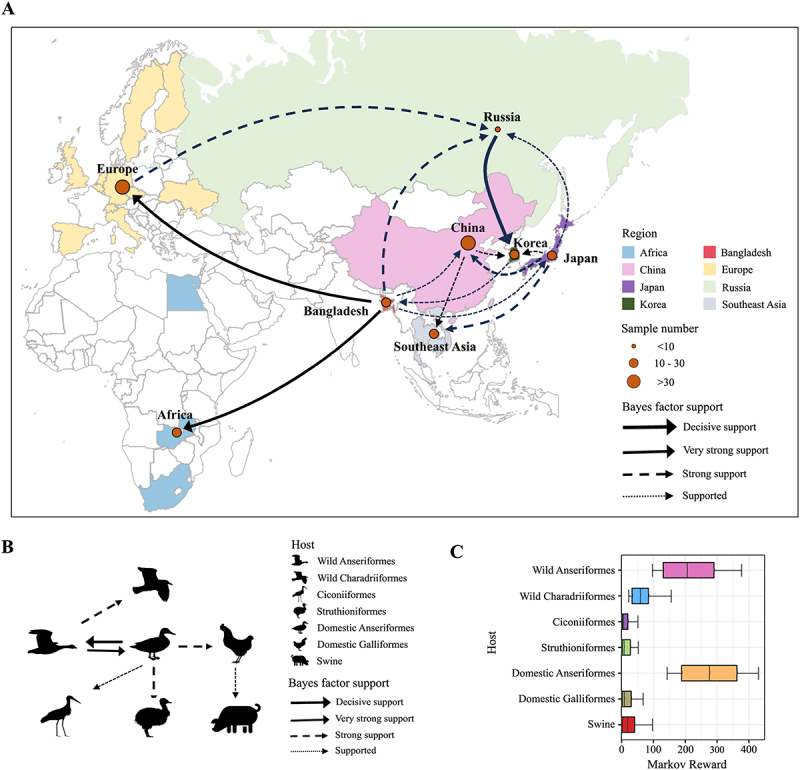
Geographic and host transmission of the Eurasian lineage H11 viruses from discrete phylogeography. (A) Spatial diffusion of Eurasian lineage H11 viruses. Colors represent different sampling regions, and circle sizes indicate the sample numbers from each region. Curved arrows denote interregional H11 virus transitions statistically supported by Bayes factors (bf) ≥ 3 and posterior probabilities (pp) ≥ 0.5, with curve widths corresponding to bf values. The bold black arrows indicate decisive support (bf ≥ 1000); solid black arrows indicate very strong support (100 ≤ bf < 1000); dashed arrows indicate strong support (10 ≤ bf < 100); and dotted arrows indicate supported diffusion (3 ≤ bf < 10). (B) Transmission patterns among different hosts of Eurasian lineage H11 viruses. Silhouettes represent various hosts. (C) Density distribution of the time H11 viruses spent in each host population (Markov rewards). The plot was generated using ggplot2 v.3.4.1.

The discrete phylogeographic analysis showed that the migration network for the Eurasian lineage H11 viruses was complex, with 13 plausible migration paths (BF ≥ 3, PP ≥ 0.5). Notably, Bangladesh was implicated in six migration pathways, indicating its central role in viral dissemination (Figure 4(A), Table S4). Japan was involved in five transmission routes, while Russia, Korea, and China were each associated with four supported migration paths. The route from Russia to Korea was identified with decisive support (BF ≥ 1000). Two routes, including Bangladesh to Europe and Bangladesh to Africa, were identified with very strong support (100 ≤ BF < 1000). Additionally, five routes with strong support were identified from Japan, Bangladesh, and Europe (Figure 4(A), Table S4). Notably, H11 viruses from Bangladesh and Japan were responsible for transmitting the virus to China, with BF of 14.62 and 4.87, respectively.

The transmission pattern of Eurasian lineage H11 viruses between different species was shown in Figure 4(B). It is speculated that domestic Anseriformes and wild Anseriformes acted as two intermediate hosts for H11 viruses to spread to other hosts (Figure 4(B), Table S5). The domestic Anseriformes were responsible for the transmission of H11 viruses to domestic Galliformes (BF = 27.95), Struthioniformes (BF = 49.36) and Ciconiiformes (BF = 8.15) (Table S5). In addition, wild Anseriformes contributed to the spillover of the viruses to wild Charadriiformes (100 ≤ BF < 1000). Furthermore, A cross-species transition from domestic Galliformes to swine was also supported by BF of 3.11. The total Markov reward time was the highest in domestic Anseriformes (275.95), followed by wild Anseriformes (206.26) (Figure 4(C), Table S6), indicating that Eurasian lineage H11 viruses had been mostly sustained among domestic Anseriformes and wild Anseriformes.

### H11 isolates from wild birds in the study underwent reassortment with clade 2.3.4.4b H5N1 HPAI viruses and established seven genotypes

According to the ML trees, the HA genes of 17 H11 isolates belonged to the Eurasian lineage, with nucleotide identities, ranging from 98.1% to 100% (Figure S2 A). The NA genes of six H11N1 isolates, with 93.2%–100% nucleotide homology, were divided into two groups, one clustered with clade 2.3.4.4b H5N1 HPAI viruses, and the other was closely related to wild bird-derived H6N1 viruses (Figure S2B). The NA genes of the H11N2 and H11N3 isolates clustered with Eurasian-lineage wild bird-origin H9N2 and chicken-origin H10N3 viruses, respectively (Figure S2C – D). Nine H11N9 isolates formed a single phylogenetic clade based on the NA genes, sharing 98.7%–100% nucleotide identity (Figure S2E). The internal gene segments of these viruses shared nucleotide identities for PB2 (93.9%–100%), PB1 (87.2%–100%), PA (94.9%–100%), NP (93.1%–100%), M (98.0%–100%), and NS (70.9%–100%) (Figure S3A – F). The PB2 genes belonged to the Eurasian lineage and were grouped into two clades. Of these, seven isolates clustered with wild bird-origin clade 2.3.4.4b H5N1 HPAI viruses, while the remaining ten grouped with wild bird-derived H4N6 viruses (Figure S3A). The PB1 genes were derived from both Eurasian and North American lineages, with the Eurasian group further subdivided into three clades (Figure S3B). The PA genes were also of Eurasian origin and clustered into three distinct groups, with two H11N9 viruses isolated in 2023 clustering with duck-derived H5N1 HPAI viruses, while the other two groups clustered with wild bird-origin H3N8 and H4N6 viruses, respectively (Figure S3C). The NP segments were distributed into three distinct phylogenetic groups (Figure S3D), while the M gene clustered into a single clade (Figure S3E), and the NS gene was classified into two clades, Allele A and Allele B (Figure S3F).

**Figure 5.**
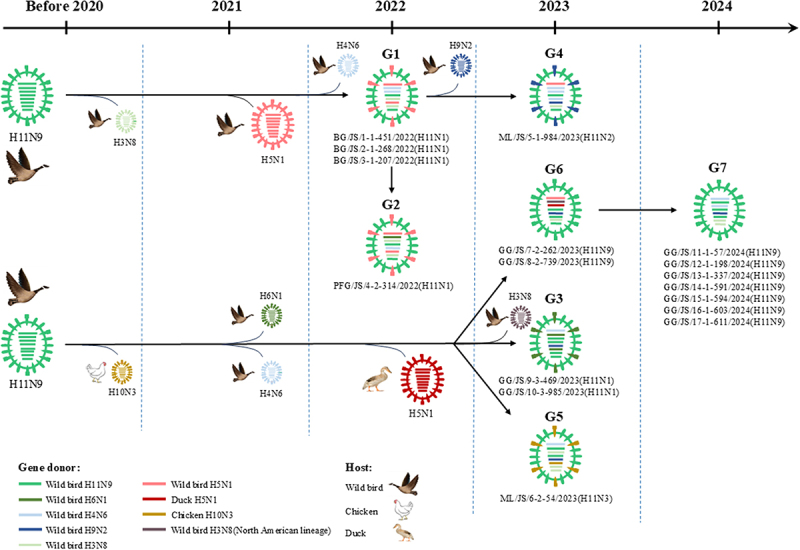
Reassortment process and genotypes of seven representative H11 isolates from wild birds. Eight gene segments of H11 isolates are represented by horizontal bars in each oval (PB2, PB1, pa, ha, np, na, M and NS, from top to bottom), colored based on their origin.

Based on phylogenetic analysis and highest nucleotide homology, the 17 H11 viruses isolated from wild birds in the study were classified into seven genotypes (G1–G7), reflecting complex reassortment events (Figure S4, Table S7). As shown in Figure 5, H11 isolates from wild birds were found to have acquired gene segments from clade 2.3.4.4b H5N1 highly pathogenic avian influenza viruses, as well as from several LPAI subtypes, including H3N8, H4N6, H6N1, H9N2, and chicken-origin H10N3. Notably, genotypes G1 and G3 shared gene segments with H5N1 HPAI viruses, underscoring the role of H11 viruses as potential genetic contributors to the evolution of HPAI strains. Overall, our findings demonstrate that H11 isolates from wild birds have experienced complex reassortments with clade 2.3.4.4b H5N1 HPAI viruses and various subtypes of LPAI viruses from wild and domestic birds.

### Molecular characteristics of the H11 isolates from wild birds in the study

The HA genes of 17 H11 isolates from wild birds in the study encoded the amino acid sequence PAIASR↓GLF at the cleavage site, suggesting that they were LPAI viruses [26]. Notably, 17 isolates exhibited S137A (H3 numbering) mutation in the HA gene (Table 2), which is associated with enhanced binding affinity of AIVs to human-type receptors [27]. Additionally, these viruses had several amino acid mutations linked to increased pathogenicity in mammals, including D3V and D622G in PB1 [28], N66S in PB1-F2 [29], and N30D, I43M, and T215A in M1 [30], as well as P42S and I106M in NS1 [31] (Table 2).

**Table 2.** Molecular markers of H11 viruses from wild birds in the study.

Virus name	Increased virus binding to human-type receptor					Increased virulence in mice and increased polymerase activity					Increased virulence in mice								
	HA (H3 numbering)^a^					PB2			PB1		NA (N2 numbering)^a^	PB1-F2	M1			M2	NS1		
	S 137 A	T 160 A	E 190 G	Q 226 L	G 228 S	D 253N	E 627 K	D 701 N	D 3 V	D 622 G	Stalk deletion	N 66 S	N 30 D	I 43 M	T 215 A	G 34 E	80–84 deletion	P 42 S	I 106 M
BG/JS/1–1-451/2022(H11N1)	A	-^b^	–	–	–	–	–	–	V	G	–	–	D	M	A	–	–	S	M
BG/JS/2–1-268/2022(H11N1)	A	–	–	–	–	–	–	–	V	G	–	–	D	M	A	–	–	S	M
BG/JS/3–1-207/2022(H11N1)	A	–	–	–	–	–	–	–	V	G	–	–	D	M	A	–	–	S	M
PFG/JS/4–2-314/2022(H11N1)	A	–	–	–	–	–	–	–	V	G	–	–	D	M	A	–	–	S	M
GG/JS/9–3-469/2023(H11N1)	A	–	–	–	–	–	–	–	V	G	–	–	D	M	A	–	–	A	M
GG/JS/10–3-985/2023(H11N1)	A	–	–	–	–	–	–	–	V	G	–	–	D	M	A	–	–	A	M
ML/JS/5–1-984/2023(H11N2)	A	–	–	–	–	–	–	–	V	G	–	–	D	M	A	–	–	S	M
ML/JS/6–2-54/2023(H11N3)	A	–	–	–	–	–	–	–	V	G	–	–	D	M	A	–	–	A	M
GG/JS/7–2-262/2023(H11N9)	A	–	–	–	–	–	–	–	V	G	–	–	D	M	A	–	–	S	M
GG/JS/8–2-739/2023(H11N9)	A	–	–	–	–	–	–	–	V	G	–	–	D	M	A	–	–	S	M
GG/JS/11–1-57/2024(H11N9)	A	–	–	–	–	–	–	–	V	G	–	–	D	M	A	–	–	A	M
GG/JS/12–1-198/2024(H11N9)	A	–	–	–	–	–	–	–	V	G	–	–	D	M	A	–	–	A	M
GG/JS/13–1-337/2024(H11N9)	A	–	–	–	–	–	–	–	V	G	–	–	D	M	A	–	–	A	M
GG/JS/14–1-591/2024(H11N9)	A	–	–	–	–	–	–	–	V	G	–	–	D	M	A	–	–	A	M
GG/JS/15–1-594/2024(H11N9)	A	–	–	–	–	–	–	–	V	G	–	–	D	M	A	–	–	A	M
GG/JS/16–1-603/2024(H11N9)	A	–	–	–	–	–	–	–	V	G	–	–	D	M	A	–	–	A	M
GG/JS/17–1-611/2024(H11N9)	A	–	–	–	–	–	–	–	V	G	–	–	D	M	A	–	–	A	M

a: The mutations/motifs are numbered according to alignments with A/Aichi/2/1968 (H3N2).

b: The “-” indicates the mutation was not detected in the H11 viruses.

### The H11 isolates from wild birds in the study exhibited dual receptor binding specificity

**Figure 6.**
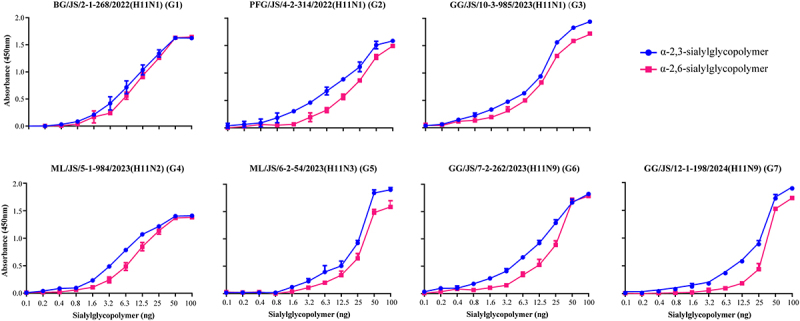
Receptor binding specificity of seven representative H11 viruses. Binding of the indicated viruses to sialylglycopolymers (α−2,3-sialylglycopolymer (avian-type receptor), blue; α − 2,6-sialylglycopolymer (human-type receptor), red). The data shown are the means of three replicates; the error represent the standard error of the mean.

Receptor binding specificity plays a critical role in host adaptation and cross-species transmission of AIVs. To further investigate the receptor binding specificity of H11 viruses isolated from wild birds, we selected seven representative viruses assessed the receptor binding affinities. As shown in Figure 6, seven representative viruses (G1–G7) exhibited dual receptor binding properties. While these viruses demonstrated a higher affinity for avian-type receptors, they also acquired the ability to bind to human-type receptors.

### The H11 isolates from wild birds in the study could infect mice directly

**Figure 7.**
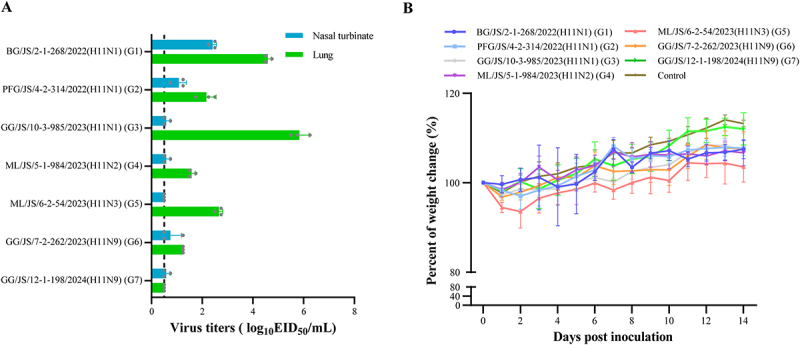
Pathogenicity and replication of seven representative H11 viruses in mice. (A) Viral titers in mouse organs on day 3 p.I. No virus was detected in the brain, spleen, or kidney, and therefore these data are not shown. The dashed line indicates the lower limit of detection. (B) Body weight changes in virus-inoculated groups (*n* = 5 per group) and the PBS-inoculated control group (*n* = 5) over a 14-day observation period. Mice were intranasally inoculated with 10^6^ EID_50_ of each virus or pbs. Error bars represent the standard deviation (sd).

Seven representative viruses of each genotype (G1–G7) were selected for replication and virulence assessments in mice. The viral titer in the nasal turbinate and lung ranged from 0.5 to 6.25 log_10_ EID_50_/mL, and no virus was detected in the spleen, kidney or brain tissue (data not shown) (Figure 7(A)). GG/JS/10–3-985/2023 (H11N1) from G3, exhibited the highest viral titer in the lungs, reaching 6.25 log_10_ EID_50_/mL. BG/JS/2–1-268/2022 (H11N1) from G1, displayed the highest viral titer in the nasal turbinate, measuring 2.42 log_10_ EID_50_/mL. As shown in Figure 7(B), the virus-inoculated groups exhibited varying degrees of transient weight loss following infection, with the lowest body weight typically observed between days 2 and 4 p.i. Notably, mice inoculated with ML/JS/6–2-54/2023 (H11N3) (G5) caused a slight body weight loss of approximately 6.5%. In contrast, control mice maintained stable weight throughout the 14-day observation period. The results indicate that the H11 isolates from wild birds could infect mice directly without prior adaptation.

## Discussion

Wild birds, particularly waterfowl and shorebirds, are recognized as the primary natural reservoirs of AIVs [10]. H11 subtype AIVs can spread worldwide by the migration of wild birds [32]. In this study, we analyzed 1,003 HA sequences of H11 viruses to investigate their distribution across time and geographic regions. The statistical results showed that H11 viruses are found on seven continents and the largest number of isolates was in North American, followed by Asia and Europe. Noteworthy, one isolate, A/swine/KU/2/2001, originated from swine, suggesting that H11 viruses possess the capacity to infect mammals. In terms of subtypes of HA and NA, H11 viruses have multiple subtype combinations (H11N1–H11N9), with H11N9 being the most frequently detected.

Bangladesh functions as a key stopover for migratory birds along several major migration routes, including the East Asia-Australia and Central Asia flyways [33]. Bayesian phylodynamic analysis of Eurasian lineage H11 viruses revealed that Bangladesh is a critical hub for the spread of H11 viruses to other regions. Additionally, migratory wild birds use both the East Asia-Australia route and the Pacific flyway via Japan, which may facilitate the transmission of various avian influenza viruses between Europe, Asia, and the Americas [34]. From a host perspective, both domestic and wild Anseriformes were found to act as important intermediate hosts in the transmission of H11 viruses to other species. Moreover, wild Charadriiformes have also been suggested as key mediators of long-distance viral spread between breeding colonies and foraging sites during migration seasons [35]. Of note, domestic Galliformes were implicated in subsequent cross-species transmission to swine.

Reassortment plays a critical role in the evolution of influenza A viruses and serves as a primary source of novel pathogenic strains [4,36]. A notable example is the emergence of the H7N9 subtype in China in 2013, which caused five waves of human infections, with a total of 1,568 cases, and the mortality was about 40% [15]. Genetic analysis revealed that the NA gene of the early H7N9 virus was derived from H11N9 viruses [37]. Between 2020 and 2021, the H5N8 viruses bearing the clade 2.3.4.4b HA gene underwent reassortment with other AIV subtypes and resulted in the emergence of multiple AIV subtypes, including H5N1, H5N2, H5N3, H5N4, H5N5, and H5N6 [38,39]. Gradually, H5N1 has become the dominant subtype responsible for avian influenza outbreaks worldwide, leading to numerous infections in mammals and domestic animals [8,40]. In this study, 17 H11 viruses isolated from wild birds were classified into seven genotypes, resulting from complex reassortment events between various LPAI viruses and clade 2.3.4.4b H5N1 HPAI viruses. Therefore, the continued recombination of H11 AIVs from wild birds deserves close public attention.

Receptor binding preference represents a critical barrier that avian influenza viruses must overcome to infect and spread to mammals or humans [36]. The HA gene of avian influenza viruses must acquire mutations that facilitate binding to human-type receptors (α 2,6-linked sialic acid) to cross the species barrier into humans. Previous studies have shown that amino acid substitutions at positions 137, 160, 190, 226, and 228 in the HA protein (H3 numbering) are crucial for changes in receptor binding specificity of avian influenza viruses [27,41]. In this study, 17 H11 isolates carried the 137A mutation, which enhances binding to human-type receptors. Moreover, seven representative viruses exhibited dual receptor-binding specificities and acquired the ability to bind to human-type receptors, revealing the potential infection of H11 AIVs to mammals.

AIVs are prone to acquiring mammalian adaptive mutations necessary for effective replication and transmission among mammals or humans [23,42]. The best-known mammalian adaptive mutations are E627K and D701N in PB2. Although none of the 17 H11 viruses in this study possessed these hallmark mutations, several mutations associated with enhanced virulence in mammals were observed, including D3V and D622G in PB1, N66S in PB1-F2, N30D, I43M, and T215A in M1, as well as P42S and I106M in NS1 [28–31,43]. The mouse model provides a practical and well-established system for evaluating the replication and pathogenicity of AIVs in mammals [44]. Animal studies have indicated that H11 isolates can infect mice directly without prior adaptation and replicate efficiently in lung and nasal turbinate tissue, causing weight loss in mice. Although genotype G3 (GG/JS/10–3-985/2023(H11N1)) displayed the highest viral titers in the lungs (6.25 log_10_ EID_50_/mL), the associated body weight loss in infected mice was less pronounced than expected. This discrepancy likely reflects viral replication restricted to the respiratory tract without systemic dissemination, together with differences in viral genetic background can decouple replication efficiency from clinical severity. Similar observations have been reported for other avian influenza subtypes, including H4N6 and H9N2, where high levels of viral replication in the lungs did not always correlate with severe weight loss or mortality in mice [7,45]. These findings provide strong evidence of the ability of H11 AIVs from wild bird to infect mammalian hosts and potentially pose a threat to public health.

In conclusion, we presented the phylogeny and biological characteristics of H11 viruses comprehensively. Our findings illustrated that H11 isolates from wild birds exhibited a high affinity for human-type receptors and infected mammalian model without prior adaptation, which could facilitate avian-to-mammals or human transmission. Therefore, it is crucial to enhance active surveillance of H11 subtype AIVs.

## Supplementary Material

TableS6.docx

TableS5.docx

TableS1.xlsx

TableS2.docx

TableS3.docx

Author Checklist Full.pdf

TableS4.docx

TableS7.docx

ARRIVE Checklist.pdf

## Data Availability

The genome sequences of 17 H11 AIVs isolated in this study were uploaded to GISAID EpiFlu™ database (Accession ID: EPI3755265 – EPI3755400) and will be made publicly accessible immediately upon acceptance of this manuscript in accordance with institutional policy. To facilitate editorial and peer review evaluation, the complete genome dataset has also been deposited in Zenodo and is available via the following private link: https://zenodo.org/records/15765458?token=eyJhbGciOiJIUzUxMiJ9.eyJpZCI6ImIzMDdjODM0LTQ0N2YtNDZkNS1hMDI5LTRlNTAxMzJlOTE3OCIsImRhdGEiOnt9LCJyYW5kb20iOiJkNDdlMTYwMTdkODExMjIzNTAzNmQ1ZDdhN2FlYzRmMCJ9.C_dXQ4jCySeoU9WXg9YRRJhNRYocGHH-gd0tCvWdSkzyuHIqvUGPRHIwhsz1Uyv3PRZU8YjX8On9KgcKfFG__w.f All other data supporting the findings of this study, including raw data from mouse infection experiments, receptor-binding assays, phylogenetic analyses, and source data for figures and tables, are openly available on Zenodo at https://doi.org/10.5281/zenodo.17211677 under a CC-BY 4.0 International license [46].
