# Supplementary material for: Epidemiology, evolution, and biological characteristics of avian influenza A (H11) viruses from wild birds
Source: Virulence. 2025 Nov 19;16(1):2591462. doi: 10.1080/21505594.2025.2591462 (PMC12645866; doi:10.1080/21505594.2025.2591462)
Supplement: TableS6.docx [file KVIR_A_2591462_SM9827.docx]

Table S6. Density distribution of the total time spent in a particular host population (Markov rewards).

| **Host** | **Mean** | **Stdev** | **Value range** | **95% HPD interval** |
| --- | --- | --- | --- | --- |
| Wild Anseriformes | 206.2573 | 42.6075 | [95.7122, 376.3647] | [130.3989, 289.9701] |
| Wild Charadriiformes | 58.1293 | 13.7044 | [21.0335, 154.954] | [32.1628, 83.549] |
| Ciconiiformes | 5.3727 | 6.1395 | [0.4667, 50.1203] | [0.6372, 19.2552] |
| Struthioniformes | 7.02 | 7.8539 | [0.4893, 51.2675] | [0.7408, 26.7996] |
| Domestic Anseriformes | 275.9502 | 45.8777 | [142.1909, 428.7592] | [188.1022, 362.3391] |
| Domestic Galliformes | 7.6044 | 9.1465 | [0.1404, 66.6685] | [0.3618, 29.6355] |
| Swine | 18.1714 | 12.313 | [0.0105, 96.0503] | [0.011, 40.1056] |
