# Supplementary material for: Epidemiology, evolution, and biological characteristics of avian influenza A (H11) viruses from wild birds
Source: Virulence. 2025 Nov 19;16(1):2591462. doi: 10.1080/21505594.2025.2591462 (PMC12645866; doi:10.1080/21505594.2025.2591462)
Supplement: TableS5.docx [file KVIR_A_2591462_SM9825.docx]

Table S5. Bayes factor of host transmission of H11 viruses in the Eurasian lineage.

| **From** | **To** | **Bays factor** | **Posterior probability** |
| --- | --- | --- | --- |
| Domestic Anseriformes | Wild Anseriformes | 47475.67427 | 1 |
| Wild Anseriformes | Domestic Anseriformes | 303.042778 | 0.98289079 |
| Wild Anseriformes | Wild Charadriiformes | 156.7759467 | 0.967448061 |
| Domestic Anseriformes | Struthioniformes | 49.36353192 | 0.903455172 |
| Domestic Anseriformes | Domestic Galliformes | 27.95162161 | 0.841239862 |
| Domestic Anseriformes | Ciconiiformes | 8.152795371 | 0.607154761 |
| Domestic Galliformes | Swine | 3.106381276 | 0.501625486 |
