# Supplementary material for: Epidemiology, evolution, and biological characteristics of avian influenza A (H11) viruses from wild birds
Source: Virulence. 2025 Nov 19;16(1):2591462. doi: 10.1080/21505594.2025.2591462 (PMC12645866; doi:10.1080/21505594.2025.2591462)
Supplement: TableS2.docx [file KVIR_A_2591462_SM9820.docx]

Table S2. Root state posterior probabilities for different geographic regions

| **Region** | **Posterior probability** |
| --- | --- |
| Africa | 0.010998778 |
| Bangladesh | 0.209532274 |
| China | 0.008332408 |
| Europe | 0.737362515 |
| Japan | 0.019442284 |
| Korea | 0.007999111 |
| Russia | 0.004332852 |
| Southeast Asia | 0.001999778 |
