# Supplementary material for: Epidemiology, evolution, and biological characteristics of avian influenza A (H11) viruses from wild birds
Source: Virulence. 2025 Nov 19;16(1):2591462. doi: 10.1080/21505594.2025.2591462 (PMC12645866; doi:10.1080/21505594.2025.2591462)
Supplement: TableS3.docx [file KVIR_A_2591462_SM9818.docx]

Table S3. Root state posterior probabilities for different host species

| **Host** | **Posterior probability** |
| --- | --- |
| Wild Anseriformes | 0.109210088 |
| Wild Charadriiformes | 0.004666148 |
| Ciconiiformes | 0.000555494 |
| Struthioniformes | 0.001333185 |
| Domestic Anseriformes | 0.87656927 |
| Domestic Galliformes | 0.001444284 |
| Swine | 0.006221531 |
