# Supplementary material for: Epidemiology, evolution, and biological characteristics of avian influenza A (H11) viruses from wild birds
Source: Virulence. 2025 Nov 19;16(1):2591462. doi: 10.1080/21505594.2025.2591462 (PMC12645866; doi:10.1080/21505594.2025.2591462)
Supplement: TableS4.docx [file KVIR_A_2591462_SM9814.docx]

Table S4. Bayes factor for spatial transmission of H11 viruses in the Eurasian lineage.

| **From** | **To** | **Bayes factor** | **Posterior probability** |
| --- | --- | --- | --- |
| Russia | Korea | 3223.20385 | 1 |
| Bangladesh | Europe | 201.7879637 | 0.961895345 |
| Bangladesh | Africa | 103.3201703 | 0.937340296 |
| Bangladesh | Russia | 23.26555633 | 0.787468059 |
| Japan | China | 14.62283936 | 0.699588935 |
| Europe | Russia | 12.32485285 | 0.662481946 |
| Japan | Southeast Asia | 11.98812781 | 0.656260416 |
| Bangladesh | Japan | 9.98908941 | 0.636373736 |
| Japan | Korea | 9.438016196 | 0.600488835 |
| Japan | Russia | 8.401087797 | 0.572269748 |
| China | Korea | 7.120303547 | 0.531385402 |
| Bangladesh | China | 4.868551715 | 0.516729252 |
| China | Southeast Asia | 6.689846622 | 0.515831574 |
| Korea | Bangladesh | 6.213970700 | 0.507389178 |
