# Supplementary material for: Epidemiology, evolution, and biological characteristics of avian influenza A (H11) viruses from wild birds
Source: Virulence. 2025 Nov 19;16(1):2591462. doi: 10.1080/21505594.2025.2591462 (PMC12645866; doi:10.1080/21505594.2025.2591462)
Supplement: TableS7.docx [file KVIR_A_2591462_SM9811.docx]

Table S7. The highest nucleotide homology of the whole genomes of seven representative H11 viruses.

| Virus  (Genotype) | | Gene | Highest homologous strain | | | Accession |
| --- | --- | --- | --- | --- | --- | --- |
|  |  |  | Name | Abbreviation | Homology |  |
| BG/JS/2-1-268/  2022(H11N1) (G1) | | HA | A/Anas poecilorhyncha/South Korea/JB32-81/2019(H11N2) | AP/South Korea/JB32-81/2019(H11N2) | 98.17% | MW116737.1 |
|  |  | NA | A/whooper swan/Henan/15/2021(H5N1) | WS/Henan/15/2021(H5N1) | 99.65% | EPI2070197 |
|  |  | PB2 | A/eurasian coot/Shandong/W22/2022(H8N4) | EC/Shandong/W22/2022(H8N4) | 99.61% | EPI2628715 |
|  |  | PB1 | A/Spot-billed duck/Korea/KNU-48/2022(H4N6) | SBD/Korea/KNU-48/2022(H4N6) | 99.65% | OR674149.1 |
|  |  | PA | A/Em/Korea/22WF191-23P/2022(H5N1) | EM/Korea/22WF191-23P/2022(H5N1) | 99.54% | EPI2742575 |
|  |  | NP | A/environment/Kagoshima/KU-H4/2021(H7N7) | EM/Kagoshima/KU-H4/2021(H7N7) | 99.20% | EPI2553081 |
|  |  | MP | A/avian/Japan/KU-d3C/2020(H2N9) | AV/Japan/KU-d3C/2020(H2N9) | 99.49% | EPI2596620 |
|  |  | NS | A/mallard/Shanghai/JDS19906/2019(H4N2) | MD/Shanghai/JDS19906/2019(H4N2) | 99.76% | EPI2600908 |
| PFG/JS/4-2-314/  2022(H11N1) (G2) | | HA | A /Anas poecilorhyncha/South Korea/JB32-81/2019(H11N2) | AP/ South Korea/JB32-81/2019(H11N2) | 98.41% | MW116737.1 |
|  |  | NA | A/whooper swan/Henan/15/2021(H5N1) | WS/Henan/15/2021(H5N1) | 99.65% | EPI2070197 |
|  |  | PB2 | A/eurasian coot/Shandong/W22/2022(H8N4) | EC/Shandong/W22/2022(H8N4) | 99.34% | EPI2628715 |
|  |  | PB1 | A/mallard/South Korea/KNU2021-46/2021(H4N6) | MD/South Korea/KNU2021-46/2021(H4N6) | 99.21% | ON513978.1 |
|  |  | PA | A/mallard/Kagoshima/KU-131/2022(H11N9) | MD/Kagoshima/KU-131/2022(H11N9) | 99.30% | EPI2794009 |
|  |  | NP | A/mallard/Kagoshima/KU-131/2022(H11N9) | MD/Kagoshima/KU-131/2022(H11N9) | 99.60% | EPI2794010 |
|  |  | MP | A/mallard/Kagoshima/KU-131/2022(H11N9) | MD/Kagoshima/KU-131/2022(H11N9) | 99.80% | EPI2794011 |
|  |  | NS | A/Mallard/Shanghai/JDS19906/2019(H4N2) | MD/Shanghai/JDS19906/2019(H4N2) | 99.64% | EPI2600908 |
| GG/JS/10-3-985/  2023(H11N1) (G3) | HA | | A/environment/Kagoshima/KU-D16/2023 (H11N9) | EM/ Kagoshima/KU-D16/2023 (H11N9) | 99.06% | EPI2917553 |
|  | NA | | A/bean goose/Korea/KNU-14/2022(H6N1) | BG/Korea/KNU-14/2022(H6N1) | 99.15% | EPI2873448 |
|  | PB2 | | A/environment/Bangladesh/52103/2022(H10N4) | EM/Bangladesh/52103/2022(H10N4) | 98.60% | EPI2201665 |
|  | PB1 | | A/common teal/Sakhalin/81c/2020(H3N8) | CT/Sakhalin/81c/2020(H3N8) | 99.65% | EPI1847533 |
|  | PA | | A/spot billed duck/Korea/KNU-30/2022(H1N1) | SBD/Korea/KNU-30/2022(H1N1) | 99.40% | EPI2873100 |
|  | NP | | A/spot billed duck/Korea/KNU-30/2022(H1N1) | SBD/Korea/KNU-30/2022(H1N1) | 99.20% | EPI2873102 |
|  | MP | | A/white fronted goose/Korea/KNU-12/2023(H1N3) | WFG/Korea/KNU-12/2023(H1N3) | 99.80% | EPI2873034 |
|  | NS | | A/wild duck/South Korea/KNU2020-31/2020(H1N1) | WD/South Korea/KNU2020-31/2020(H1N1) | 99.64% | EPI1931606 |
| ML/JS/5-1-984/  2023(H11N2) (G4) | HA | | A/Anas poecilorhyncha/South Korea/JB32-81/2019(H11N2) | AP/South Korea/JB32-81/2019(H11N2) | 97.94% | MW116737.1 |
|  | NA | | A/spot billed duck/Korea/KNU-28/2022(H9N2) | SBD/Korea/KNU-28/2022(H9N2) | 99.79% | EPI2873115 |
|  | PB2 | | A/eurasian coot/Shandong/W22/2022(H8N4) | EC/Shandong/W22/2022(H8N4) | 99.43% | EPI2628715 |
|  | PB1 | | A/spot-billed duck/Korea/KNU-48/2022(H4N6) | SBD/Korea/KNU-48/2022(H4N6) | 99.56% | OR674149.1 |
|  | PA | | A/Em/Korea/22WF191-23P/2022(H5N1) | EM/Korea/22WF191-23P/2022(H5N1) | 99.44% | EPI2742575 |
|  | NP | | A/eurasian eagle owl/Korea/22WC032/2022(H5N1) | EGO/Korea/22WC032/2022(H5N1) | 99.06% | EPI2742476 |
|  | MP | | A/avian/Japan/KU-d3C/2020(H2N9) | AV/Japan/KU-d3C/2020(H2N9) | 99.59% | EPI2596620 |
|  | NS | | A/mallard/Shanghai/JDS19906/2019(H4N2) | MD/Shanghai/JDS19906/2019(H4N2) | 99.40% | EPI2600908 |
| ML/JS/6-2-54/  2023(H11N3) (G5) | HA | | A/environment/Kagoshima/KU-D16/2023 (H11N9) | EN/Kagoshima/KU-D16/2023 (H11N9) | 99.35% | EPI2917553 |
|  | NA | | A/chicken/China/HAUST-16/2023(H3N3) | CK/China/HAUST-16/2023(H3N3) | 99.65% | EPI2731197 |
|  | PB2 | | A/anser albifrons/South Korea/163-5/2022(H10N7) | AB/South Korea/163-5/2022(H10N7) | 99.17% | EPI2395065 |
|  | PB1 | | A/Zhejiang/CNIC-ZJU01/2023(H10N5) | ZJ/CNIC-ZJU01/2023(H10N5) | 99.34% | EPI2970866 |
|  | PA | | A/duck/Bangladesh/19D2183/2023(H9N2) | DK/Bangladesh/19D2183/2023(H9N2) | 98.93% | EPI3116096 |
|  | NP | | A/environment/Kagoshima/KU-G10/2022 (H10N4) | EM/Kagoshima/KU-G10/2022(H10N4) | 99.20% | EPI2553169 |
|  | MP | | A/white-fronted goose/South Korea/KNU2021-18/2021(H6N2) | WFG/South Korea/KNU2021-18/2021(H6N2) | 99.80% | EPI2153859 |
|  | NS | | A/wild duck/South Korea/KNU2020-31/2020(H1N1) | WD/South Korea/KNU2020-31/2020(H1N1) | 99.64% | EPI1931606 |
| GG/JS/7-2-262/  2023(H11N9) (G6) | HA | | A/environment/Kagoshima/KU-D16/2023 (H11N9) | EM/Kagoshima/KU-D16/2023 (H11N9) | 99.29% | EPI2917553 |
|  | NA | | A/white fronted goose/Korea/KNU-01/2023(H11N9) | WFG/Korea/KNU-01/2023(H11N9) | 98.65% | EPI2873051 |
|  | PB2 | | A/environment/Japan/KU-4h/2021(H3N8) | EM/Japan/KU-4h/2021(H3N8) | 99.39% | EPI2596070 |
|  | PB1 | | A/barnacle goose/Germany-NI/2023AI08854/2023(H9N2) | BG/Germany-NI/2023AI08854/2023(H9N2) | 99.56% | EPI2904450 |
|  | PA | | A/chicken/Korea/H124/2022(H5N1) | CK/Korea/H124/2022(H5N1) | 98.42% | EPI2540660 |
|  | NP | | A/Goose/Korea/H277/2022(H5N3) | GS/Korea/H277/2022(H5N3) | 98.66% | EPI2113372 |
|  | MP | | A/white-fronted goose/South Korea/KNU2021-18/2021(H6N2) | WFG/South Korea/KNU2021-18/2021(H6N2) | 99.80% | EPI2153859 |
|  | NS | | A/peregrine falcon/Saga/4112A002/2023(H5N6) | PF/Saga/4112A002/2023(H5N6) | 99.88% | EPI2898981 |
| GG/JS/12-1-198/  2024(H11N9) (G7) | HA | | A/environment/Kagoshima/KU-D16/2023 (H11N9) | EM/Kagoshima/KU-D16/2023(H11N9) | 99.00% | EPI2917553 |
|  | NA | | A/white fronted goose/Korea/KNU-01/2023(H11N9) | WFG/Korea/KNU-01/2023(H11N9) | 99.22% | EPI2873051 |
|  | PB2 | | A/environment/Bangladesh/52103/2022(H10N4) | EM/Bangladesh/52103/2022(H10N4) | 98.73% | EPI2201665 |
|  | PB1 | | A/mallard/South Korea/KNU2021-17/2021(H3N8) | ML/South Korea/KNU2021-17/2021(H3N8) | 98.90% | EPI2153469 |
|  | PA | | A/spot-billed duck/South Korea/KNU2022-73/2022(H4N6) | SBD/South Korea/KNU2022-73/2022(H4N6) | 99.16% | EPI3268063 |
|  | NP | | A/spot-billed duck/Korea/KNU-30/2022(H1N1) | SBD/Korea/KNU-30/2022(H1N1) | 98.93% | EPI2873102 |
|  | MP | | A/white fronted goose/Korea/KNU-12/2023(H1N3) | WFG/Korea/KNU-12/2023(H1N3) | 99.59% | EPI2873034 |
|  | NS | | A/wild duck/South Korea/KNU2020-31/2020(H1N1) | WD/South Korea/KNU2020-31/2020(H1N1) | 99.64% | EPI1931606 |
